# Gender Roles in Chinese Children’s Picture Books: A Descriptive Content Analysis

**DOI:** 10.3390/bs16081312

**Published:** 2026-08-02

**Authors:** Junnan Zhou, Jiahui Guo, Shuo Liu, Qi Sun, Chenyi Zhang

**Affiliations:** 1 Faculty of Education, Tianjin Normal University, Tianjin 300387, China; 2411350447@stu.tjnu.edu.cn (J.G.); 2411350446@stu.tjnu.edu.cn (S.L.); 2 Department of Educational and Counseling Psychology, School of Education, University at Albany, State University of New York, Albany, NY 12222, USA; qisunnantong@gmail.com; 3 Department of Early Childhood and Elementary Education, College of Education and Human Development, Georgia State University, Atlanta, GA 30303, USA; czhang15@gsu.edu

**Keywords:** Chinese-authored picture books, non-Chinese-authored picture books, gender roles, content analysis

## Abstract

Early childhood is a critical period for gender-role development, and picture books are one of the important mediums through which children encounter social expectations about gender. However, limited research has compared gender roles in Chinese-authored and non-Chinese-authored picture books. This study conducted a content analysis of 148 picture books selected from lists recommended by the Ministry of Education of China, award-winning Chinese-authored picture books, and the iRead Early Childhood Reading Catalog. Gender roles were examined across cover characters, protagonists, physical appearance, activities, social identity, and behavioral characteristics. Both groups displayed gender stereotypes to varying degrees. Male characters appeared more frequently on covers and as protagonists, and the difference between the two groups were not statistically significant. Regarding behavioral characteristics, male characters were significantly overrepresented in exploratory action in both book categories. No behavioral category in either book type showed a significant overrepresentation of female characters. Despite some efforts to challenge conventional portrayals, both groups largely reflected traditional gender frameworks and binary understandings of gender. These findings highlight the need for picture book authors, parents, and educators to critically examine gender roles in children’s literature and to provide children with more diverse and equitable models of gender development.

## 1. Introduction

Children’s picture books tell stories through the integrated interplay of illustrations and text (Peng, 2007). Their visual immediacy and narrative accessibility make them popular with young children and families (Sun & Wang, 2013). As cultural media, picture books present recurring character types, behaviors, and role expectations. These representations may provide children with identity templates and gender-role models (Jin & Yang, 2021; Narahara, 1998). Prior research suggests that children may observe different behaviors, encounter diverse identities, and develop an understanding of lifestyles different from their own (Götz et al., 2005). As a sociocultural norm, a gender role comprises behavioral expectations associated with an individual’s gender and acquired through imitation and learning during socialization (Fisher et al., 2016). Gender roles represented in picture books are shaped by the sociocultural contexts in which the books are created and circulated (Hentschel et al., 2019). Existing research has documented gender inequality and limited gender diversity in children’s literature across multiple cultural contexts (Adams et al., 2011; Anderson & Hamilton, 2005). For instance, Matta et al. (2026) conducted a comparative study of Hebrew and Arabic picture books, revealing significant disparities in gender role representation among books from different linguistic and cultural backgrounds within the same country. Yeoh and Cheong (2025) further noted that children’s literature written by Western authors may reinforce specific gender stereotypes when used in non-Western contexts. Because children’s picture books are important educational resources for literacy and moral education in China, this study conducted a content analysis to investigate the gender roles in Chinese children’s picture books with consideration of the authors’ background, i.e., Chinese authors vs. non-Chinese authors.

### 1.1. Theoretical Framework for Gender Representation in Picture Books

Vygotsky’s (1978) sociocultural theory states that child development is not an isolated process of individual construction but is rooted in specific sociocultural contexts and realized through social interaction and the use of cultural tools, including language and symbolic representation. From this perspective, picture books function as a particular kind of cultural tool: they make prevailing gender norms, roles, and value judgments available to children through visual and textual signs, and they do so within an inherently social activity. During parent–child shared reading and group read-alouds in kindergarten classrooms, adults further mediate these meanings through questioning, guidance, and discussion, shaping how the content of a book is interpreted rather than simply transmitting it unaltered (P. Y. Chen, 2024; Davenport et al., 2025; Yigit-Gencten et al., 2024). This mediated, socially embedded process of meaning-making underscores the importance of the content of picture books. The content created by authors set the terms of what that parent–child interaction involves.

Whereas sociocultural theory explains why picture book content matters as a transmitted cultural resource, gender schema theory offers a framework for understanding how such content may be organized and interpreted once encountered. This perspective holds that recurring cues concerning physical appearance, activities, social identities, and behavioral characteristics can be organized into gender-related categories that guide the interpretation of characters (Frawley, 2008). Young children, in turn, may apply a boy/girl framework as an interpretive lens when identifying and understanding characters (Hill & Bartow Jacobs, 2020). Because this schema-based processing tends to emerge early and operates on precisely the kinds of surface cues that authors control, such as how a character looks, what a character does, and how a character behaves toward others, gender schema theory provides a principled basis for identifying which features of a text are likely to function as gender cues in the first place. In the present study, this perspective is used specifically to inform the identification of observable gender cues within the coding system; it is not used to support claims about how individual children respond to or are affected by particular books. This distinction is important: gender schema theory explains the interpretive relevance of certain textual and visual features, not the psychological outcomes of exposure to them.

At the cultural level, Schwartz’s (2006) theory of cultural value orientations provides a complementary interpretive lens for understanding what gendered patterns of representation might signify beyond individual character traits. Two dimensions are particularly relevant. The autonomy–embeddedness dimension concerns whether individuals are represented as independent agents pursuing their own goals or as members defined primarily by collective roles and obligations. The egalitarianism–hierarchy dimension concerns whether roles, authority, and resources are distributed equally among characters or according to ascribed status. Together, these dimensions support the interpretation of differences in characters’ agency, family responsibilities, occupational roles, and positions of authority. Consistent with the study’s descriptive aims, these dimensions are used interpretively to make sense of observed patterns of representation.

Taken together, these three perspectives jointly informed both the coding and interpretation of the present study. Character appearance and narrative centrality were coded to assess gender visibility; physical appearance captured visual gender cues; and activities, social identities, and behavioral characteristics captured patterns of agency, caregiving, occupation, authority, and role expectations. Consistent with the boundaries each theory sets on its own use, the resulting codes describe patterns of representation within the book corpus itself, rather than children’s internalization of or responses to those representations.

### 1.2. Representation of Gender Roles in Children’s Literature

A substantial body of research documents persistent gender imbalance in the frequency with which male and female characters appear in picture books. Male characters are consistently overrepresented relative to female characters in title roles, as protagonists, and in illustrations more broadly (Casey et al., 2021; Hamilton et al., 2006; Lindsay, 2024; Tepper & Cassidy, 1999; Turner-Bowker, 1996; M. Wang & Lee, 2024; Weitzman et al., 1972). This asymmetry in visibility has been treated in the literature not merely as a numerical artifact but as an early indicator of whose experiences are positioned as narratively central.

A line of research examines how visual elements such as clothing and body type convey gender information. Female characters are frequently depicted as young, beautiful, slender, and delicately presented, wearing feminine attire such as pink clothing, lace, skirts, or dresses, with accessories dominated by bows, jewelry, and headwear that emphasize physical attractiveness (Foster, 2014; Harriger et al., 2010; Kortenhaus & Demarest, 1993). Male characters, by contrast, are more often rendered as strong and rugged, in clothing dominated by dark colors, simple styles, and trousers, with decorative accessories rarely present; this visual coding places greater emphasis on power and agency than on appearance (Kortenhaus & Demarest, 1993; Nair & Talif, 2010; Turner-Bowker, 1996; Weitzman et al., 1972). These findings suggest that gendered visual appearance operates as a distinct channel of representation, one that conveys social meaning independently of a character’s actions or dialogue.

Gender differentiation is also evident in the spatial settings and activity types assigned to characters. Male characters tend to appear more frequently in outdoor settings, while female characters are more often confined to indoor spaces (Hamilton et al., 2006; Tognoli et al., 1994; M. Wang & Lee, 2024). This spatial pattern is echoed in research on the gendered representation of material culture, which finds that female characters’ activity spaces are largely limited to the domestic sphere, whereas male characters are more active in public, productive spaces beyond the home (Crabb & Bielawski, 1994). A parallel pattern holds for activity type. For example, female protagonists engage in nurturing activities at a significantly higher rate than male protagonists, while male characters are afforded a broader and more varied range of activities overall (Hamilton et al., 2006).

Gender differentiation extends to how characters’ identities are positioned within family and occupational structures. Within the family, mothers are more frequently depicted assuming nurturing and caregiving responsibilities, while fathers are more often positioned as companions and material providers. This pattern has been characterized as a binary framework of the “emotional mother” and the “instrumental father” (DeWitt et al., 2013; Liu et al., 2026). In occupational contexts, male characters are represented across a more diverse range of professions, whereas female characters are more likely to be shown performing unpaid labor or to appear solely within a familial capacity (Hamilton et al., 2006; Hendricks et al., 2010; Lindsay, 2024; M. Wang & Lee, 2024). Even when female characters are assigned an explicit profession, these roles tend to be confined to traditionally feminized occupations such as teaching or nursing.

These patterns of role and setting are accompanied by corresponding differences in behavior and personality attribution. Female characters’ behaviors are largely confined to nurturing, housework, and emotional expression, while male characters are more frequently shown engaging in exploration, problem-solving, and adventurous action (Hamilton et al., 2006). Corresponding trait attributions follow a similar logic: male characters are predominantly characterized as brave, proactive, adventurous, independent, and assertive, while female characters are more often characterized as passive, quiet, emotional, gentle, and oriented toward caregiving (N. Chen, 2012; Pownall & Heflick, 2023; Qiao & Wang, 2017; Turner-Bowker, 1996; Weitzman et al., 1972).

Collectively, existing studies indicate that gender bias and stereotyping remain pronounced in children’s literature across a range of cultural contexts (Casey et al., 2021; Haghanikar et al., 2022; Huang et al., 2025). Comparative studies reinforce this conclusion while also revealing culturally specific variation. Roberts et al. (2023), for example, found that the United States, Czech Republic, and Spain each exhibit conservative tendencies in the portrayal of gender roles, with Czech picture books showing the strongest inclination toward traditional role depictions. Li et al. (2023) found that both UK and Chinese best-selling picture books overrepresent male characters and position female characters in subordinate status, with gender-stereotyped roles and traditional family structures embedded more deeply within the Chinese best-sellers examined. Similarly, Crawfurd et al. (2024) found that across textbooks from 34 countries, female characters were generally underrepresented and disproportionately associated with family and appearance, while male characters were more strongly associated with work and achievement. These recurring patterns across independent studies reflect what Blumberg (2008) described as a “striking consistency” in the underrepresentation of women and the persistence of gender stereotypes across both Western and non-Western contexts.

### 1.3. Authorial Cultural Positioning

While it is established that gendered patterns recur across picture books, comparatively few studies investigate why authorial cultural positioning might matter to how children engage with them. For example, picture books as cultural tools do not transmit meaning in the abstract, but depends on their fit with the specific sociocultural context in which a child is developing (Vygotsky, 1978). An author writing from within a given cultural context is, by virtue of that positioning, drawing on the same repertoire of social categories, family structures, and role expectations that the child reader is already encountering through daily life (Adam & Harper, 2023; Gu & Catalano, 2022; Yeoh & Cheong, 2025). Take, parental figures in picture books, for example. In picture books created by Chinese authors, gender roles tend to follow a traditional division of labor, with fathers cast as economic providers and mothers confined to domestic caregiving; male characters are also more frequently accorded central narrative status (N. Chen, 2016; N. Chen & Zhao, 2018). In picture books created by American authors, father figures are conventionally depicted as breadwinners and playmates, while mothers assume nurturing and caretaking responsibilities; female characters, moreover, appear significantly less often than male characters in titles, as protagonists, and in illustrations (Cutler & Lewis, 2024; Hamilton et al., 2006). In picture books created by British authors, the mother figure, while granted a “civilizing” narrative role, remains situated within a postfeminist ideology of “new momism” that confines her agency primarily to the domestic and emotional domains (Joosen, 2015). This suggests that gender-role depictions authored by writers embedded in Chinese cultural contexts may encode role expectations, family structures, and behavioral norms in ways that are more culturally coherent and familiar to Chinese child readers than depictions authored outside that context and subsequently translated. If so, such depictions may be comprehended and reconstructed more readily by children, not because the gender roles portrayed are inherently simpler, but because they align more closely with the interpretive schema the child is already building through everyday sociocultural experience (Frawley, 2008; Hill & Bartow Jacobs, 2020).

Non-Chinese-authored picture books, by contrast, originate from an authorial standpoint shaped by a different cultural value system. Even where translation preserves narrative content faithfully, the underlying gender-role logic embedded by the original author reflects the value orientations of the source culture rather than the receiving one. Under Schwartz’s (2006) framework, this raises the possibility that non-Chinese-authored works may present children with autonomy–embeddedness or egalitarianism–hierarchy configurations that diverge from those prevailing in their immediate sociocultural environment. In other words, picture books created by Chinese authors and translated books created by non-Chinese authors may differ systematically in the cultural coherence of the gender-role models they present, which is itself a meaningful dimension for comparison rather than an incidental feature of the corpus. Comparing pictures books by Chinese and non-Chinese-authored picture books allows the present study to examine not only what gender roles are represented, but whether the cultural positioning of the author corresponds to systematic differences in how those roles are constructed.

### 1.4. Current Study

Although existing research has documented gender-role imbalances in picture books in China and elsewhere, work situated specifically within the Chinese context remains comparatively limited. This gap is particularly consequential given that the Chinese children’s book market imports a substantial volume of foreign picture books (Yuan & Wang, 2018). As a result, non-Chinese-authored picture books have become an important cultural intermediary shaping the gender socialization of children in China. The existing literature nonetheless lacks systematic comparison of how gender roles are represented in Chinese-authored versus non-Chinese-authored picture books. To address this gap, the present study compared gender-role patterns across these two groups and provides empirical evidence for understanding gender construction in children’s literature within the Chinese context. The present study addresses the following research questions:

(1) How is gender represented in Chinese-authored picture books compared with those non-Chinese-authored picture books? (a) Are there statistically significant differences between the two groups in the gender distribution of cover characters and protagonists? (b) What descriptive patterns characterize the physical appearance of male and female characters in Chinese-authored versus those non-Chinese-authored picture books?

(2) What descriptive patterns can be identified in the activities and social identities of male and female characters in Chinese-authored picture books compared with those non-Chinese-authored picture books?

(3) Are there differences in the behavioral characteristics associated with male and female characters between Chinese-authored and non-Chinese-authored picture books?

## 2. Methods

### 2.1. Sample Selection and Screening Procedure

Picture books were selected from three sources. First, the official recommended reading list for children developed and published by the Ministry of Education of the People’s Republic of China (2021), which reflects national reading policy orientations and educational values. Second, award-winning books from nationally recognized Chinese children’s picture-book awards, such as the Feng Zikai Children’s Picture Book Award, Hsin Yi Picture Book Award, China Original Picture Book Era Award, and Bronze Sunflower Picture Book Award. This source reflects the professional artistic standards of Chinese-authored picture books (Z. Wang & Liu, 2017). Third, the iRead Early Childhood Reading List (developed by the Shenzhen iRead Foundation), which highlights developmental appropriateness and practical guidance for early reading. Together, these sources cover policy recommendation, professional evaluation, and practical early-reading use, thereby broadening the scope of the sample. Their age-related classifications also support comparability in content difficulty and narrative form.

In this study, Chinese-authored picture books were defined as works written by Chinese authors (e.g., *That Deep Blue Bird Is My Daddy, Shopping at the Bazaar with Dad,* and *A New Year’s Reunion*). Non-Chinese-authored picture books were defined as works written by authors of other nationalities and subsequently translated into Chinese (e.g., *The Tiger Who Came to Tea, Mitchell’s License,* and *Ella Sarah Gets Dressed*). This classification was based on the nationality of the authors rather than the place of first publication.

The sample selection process began with 347 picture books on the Ministry of Education of China’s recommended reading list. In the first round of screening, books featuring animal protagonists (*n* = 102), popular science titles (*n* = 110), and books without clearly identifiable gender roles (*n* = 75) were excluded. Titles could meet multiple exclusion criteria, but each was excluded only once and counted under its primary category. This process yielded 50 Chinese-authored picture books and 10 non-Chinese-authored picture books. The study further expanded the sampling sources by including award-winning picture books published between the establishment of each award and 2024, and titles from the most recent available version of the iRead Early Childhood Reading List. The award lists contained 236 published Chinese-authored picture books. After excluding books featuring animal protagonists (*n* = 59), popular science titles (*n* = 41), and books without clearly identifiable gender roles (*n* = 95), 41 Chinese-authored picture books remained. Of these, 12 were duplicates of books identified in the first-stage sample, resulting in 29 additional Chinese-authored picture books and a total of 79. The iRead list contained 239 non-Chinese-authored picture books. After excluding books featuring animal protagonists (*n* = 48), popular science titles (*n* = 50), and books without clearly identifiable gender roles (*n* = 78), 63 non-Chinese-authored picture books remained. Of these, four were duplicates of titles identified in the first-stage sample, resulting in 59 additional non-Chinese-authored picture books and a total of 69. After the samples from all three sources were combined, the final corpus comprised 79 Chinese-authored and 69 non-Chinese-authored picture books, for a total of 148 books included in the content analysis of gender-role representation (See Appendix A).

### 2.2. Coding Scheme and Category Definitions

Content analysis is a research method for analyzing texts and describing and interpreting the written artifacts of a society (White & Marsh, 2006). This study adopted content analysis (Krippendorff, 2004) and used QSR NVivo 15.0 qualitative analysis software to conduct systematic coding and analysis of the text and images in picture books. The coding dimensions include cover characters, protagonists, physical appearance, character activities, social identity, and behavioral characteristics (see Table 1). The book was the sampling unit, whereas the coding unit varied across dimensions. Cover characters and protagonists were coded once at the book level. Physical appearance was coded for each identifiable human character, activities and social identities were coded for each distinct activity or role attributed to a character, and behavioral characteristics were coded as explicit behavioral events. Each dimension was coded independently. When a character displayed multiple features, roles, activities, or behaviors, all applicable categories were coded. Therefore, the totals vary across dimensions and may exceed the number of books or characters. Only information explicitly presented in the illustrations or text was coded; characteristics inferred without observable evidence were excluded.

The coding dimensions were established with reference to previous studies and adapted to the aims of the present research. The coding of cover characters and protagonists was based primarily on the categories proposed by Ji and Zhang (2021). The physical appearance dimension was adapted from Chu (2020) and included clothing style, hairstyle, and carried items; clothing color was additionally classified as high or low lightness to reflect the visual characteristics of the sampled books. Character activities were also coded following Chu’s (2020) classification. Occupational identity was based mainly on Ji and Zhang (2021), whereas family identity drew on Lu’s (2024) classification, with educator added as an additional category. Finally, because observable behavior is an important means of character construction and value expression in picture books, four behavioral categories—emotional interaction, exploratory action, collaborative support, control and dominance—were incorporated to meet the analytical needs of this study.

#### 2.2.1. Patterns of Gender Representation

Cover characters coding focused on the content presented on the picture book covers. Each cover was classified as featuring a male character, a female character, both male and female characters, or no identifiable gender feature. Examples include a male character in *An Zai Will Get Better*, a female character in *Diandian’s Summer*, male and female characters together in *A New Year’s Reunion*, and no identifiable gender feature in *Teeth, Teeth, Throw Them on the Roof*. The last category does not refer to gender-neutral human characters but to covers featuring animals, toys, vehicles, or other non-human or inanimate subjects.

Protagonists were identified according to plot centrality, narrative perspective, and narrative weight and were classified as male, female, both male and female, or no identifiable gender feature protagonist. Examples include a male protagonist in *An Zai Will Get Better*, a female protagonist in *Diandian’s Summer*, male and female co-protagonists in *Water!*, and no identifiable gender feature protagonist in *Where Does Rice Come From?*. When group narration was used or no central character could be identified, the book was coded in the final category.

Physical appearance focuses on the character’s clothing, hairstyle and carried items. Among them, the clothing dimension includes style and color. Clothing style was coded as trousers or skirts. Trousers included jumpsuits, work clothes, and other garments without a skirted hem, whereas skirts included skirts and dresses. Clothing color was coded according to visual lightness as low-lightness or high-lightness. Low-lightness colors included black, dark gray, dark blue, and dark brown, whereas high-lightness colors included orange, pink, yellow, and light blue. For example, in *Xiaodi and the Tearful Orange*, the mother’s black dress was coded as low lightness, whereas her orange dress was coded as high lightness. The hairstyle dimension was coded as long hair when the hair reached or extended below the shoulders and as short hair when it remained above the shoulders. Carried items refer to items that the character actively carries or uses and were classified by function as tools (such as hammers, glasses, canes, binoculars and other items with functional uses), toys (such as dolls, balls, building blocks and other items), decorations (such as hairpins, hats, bags, rings and other items), or household items (such as aprons, spatulas, rags, brooms and other items). For example, the flashlight and shovel were coded as tools in *Diandian’s Summer*; the warrior figure was coded as a toy in *“Baby, I Understand You” Picture Book Series: The Amazing Luo En*; the watch was coded as a decoration in *I Won’t Give Up My Rubber Band*; and the apron, plate, and cleaning cloth were coded as household items in *I Can Explain*. Items merely present in the setting but not carried, worn, or used by the character were not coded.

#### 2.2.2. Activities and Social Identity

Character activities focus on activity settings and activity types. Activity settings were categorized as indoor or outdoor according to the location in which the focal action occurred. Indoor settings included enclosed spaces such as homes, classrooms, and rooms, whereas outdoor settings included parks, streets, fields, and other open spaces. Activity types were classified as work (occupational or job-related tasks), housework (domestic tasks such as cleaning, cooking, and organizing), social interaction (communication, cooperation, play, or helping), leisure (recreational or resting activities), or learning (reading, writing, or attending lessons). Illustrative examples included medical treatment in *I’m Not Afraid of Shots* (work), tidying toys and books in *Nana Cleans the Room* (housework), interacting with others in *My Friend with Autism* (social interaction), having afternoon tea in *The Tiger Who Came to Tea* (leisure), and writing an essay in *Yiwazi* (learning). A single activity could receive more than one code if it clearly met the criteria for multiple categories.

Social identity includes family identity and occupational identity. Family identity encompasses educator, housework laborer, economic provider, and emotional supporter. The educator imparts knowledge, guides behavior, or transmits family and cultural knowledge, as when a grandfather explains New Year traditions in *Ringing Out the Old Year and Welcoming the New: Spring Festival*. The housework laborer undertakes domestic tasks or personal care, as illustrated by the father rinsing rice and the mother trimming vegetables in *Summer Night Concert*. The economic provider supplies financial income or material resources, as the father works in *A Small One, A Big One*. The emotional supporter provides comfort, companionship, or psychological support, as when the father holds Xiaodi’s hand in *Xiaodi and the Tearful Orange*. Occupational identity refers to specialized roles within the occupational structure, such as police officers, doctors, nurses, teachers, and merchants. An occupation was coded only when it was explicitly named in the text or clearly indicated through the character’s clothing, workplace, and job-related actions; merely appearing in a workplace was insufficient.

#### 2.2.3. Behavioral Characteristics

Behavioral characteristics focus on embodied behavioral expressions and are classified into emotional interaction, exploratory action, collaborative support, control and dominance. Emotional interaction refers to explicit interpersonal expressions of affection, comfort, or emotional support through language or bodily action, such as the father lifting the child onto his shoulders in *A New Year’s Reunion*. Facial expressions or emotions not directed toward another character were not coded as interaction. Exploratory action includes curiosity-driven investigation of the environment or physically dynamic actions requiring initiative and mobility, such as climbing a table in *A Journey of 9000 Millimeters.* Routine walking or movement without exploratory intent or physical challenge was excluded. Collaborative support refers to coordinated assistance, task sharing, or complementary action directed toward a common goal, such as the maintenance worker repairing the traffic light while the police officer directs traffic in *Sid the Signal*. Mere co-presence or parallel activity without coordination was not coded as collaboration. Control and dominance refer to initiating an activity, formulating a plan, directing others, controlling a situation, or determining the course of events. For example, *in Summer Night Concert*, the father proposes the concert, assigns activities, and announces its conclusion. Simply speaking, participating, or holding an adult status was not sufficient for coding dominance.

#### 2.2.4. Reliability

Two trained research staff independently coded all 148 picture books (79 Chinese-authored and 69 non-Chinese-authored picture books) in accordance with the Coding Scheme. Inter-coder reliability was assessed using the book as the unit of analysis. For cover characters and protagonists, agreement concerned the category assigned to each book; for dimensions permitting multiple codes, each code was assessed within each book. Agreement was reviewed after each batch of 10 books as an interim quality-control procedure. If the standard was not met, the coders received further calibration and recoded the relevant books. Differences in coding decisions were resolved through discussion and, when necessary, consultation with a third researcher. The final Cohen’s kappa values were calculated from a randomly selected 20% of the corpus. All dimensions showed substantial inter-coder agreement, with Cohen’s κ values exceeding 0.90 (McHugh, 2012). Specifically, Cohen’s κ was 0.95 for cover characters and protagonists, 0.98 for physical appearance, 0.95 for character activities, 0.98 for social identity, and 0.97 for behavioral characteristics (all *p* < 0.001).

## 3. Results

### 3.1. Gender Representation Patterns in Chinese Children’s Picture Books

#### 3.1.1. Gender Distribution of Cover Characters and Protagonists

Cover and protagonists’ results were summarized as frequencies and percentages at the book level. As shown in Table 2, male characters were more frequently depicted as the sole character on the cover than female characters in both categories of picture books. A chi-square test of independence was conducted to examine whether the distribution of cover character gender categories differed between Chinese-authored and non-Chinese-authored picture books. The association between book type and cover character category was not significant, χ^2^ (3) = 2.51, *p* = 0.474, Cramer’s V = 0.13. Because some expected counts were relatively small, Fisher’s exact test with Monte Carlo simulation was also conducted and produced the same conclusion, *p* = 0.490. Thus, Chinese-authored and non-Chinese-authored picture books did not significantly differ in the overall distribution of the four cover character categories: male character, female character, both male and female present, and no identifiable gender feature. Descriptively, male characters accounted for a larger proportion of cover characters in the non-Chinese-authored picture books. However, because the difference between the two groups was not statistically significant, this pattern should not be interpreted as evidence of a stronger male-centered tendency in non-Chinese-authored picture books. Nevertheless, the within-corpus proportions indicated some imbalance in the gender distribution of cover characters.

Additionally, some picture-book covers presented non-human subjects rather than gendered human characters. For instance, the cover of the picture book *Teeth, Teeth, Throw Them on the Roof* features no human character with identifiable gender markers; instead, the old Nanjing rooftops and an activity scene formed the cover’s main visual focus.

As shown in Table 3, there is a gender difference in protagonist distribution across both categories of picture books, with male protagonists appearing more frequently than female protagonists. A chi-square test of independence was conducted to examine whether the distribution of protagonist gender categories differed between Chinese-authored and non-Chinese-authored picture books. The association between book type and protagonist gender category was not significant, χ^2^ (3) = 5.81, *p* = 0.121, Cramer’s V = 0.20. Because one expected cell count was below 5, Fisher’s exact test with Monte Carlo simulation was also conducted and produced the same conclusion, *p* = 0.127. Thus, Chinese-authored and non-Chinese-authored picture books did not significantly differ in the overall distribution of protagonist gender categories.

#### 3.1.2. Physical Appearance in Chinese-Authored and Non-Chinese-Authored Books

Physical appearance was analyzed using three categories: clothing, hairstyle, and carried items (Table 4). In both categories of picture books, male characters are predominantly depicted in trousers, with short hair and low-lightness clothing (i.e., darker and deeper hues such as brown, dark blue and dark gray); female characters, by contrast, are mainly shown in dresses, with long hair and high-lightness clothing (i.e., lighter and brighter shades such as red, orange and yellow). In Chinese-authored picture books, the proportion of female characters with long hair was considerably higher than that of female characters with short hair, while in non-Chinese-authored picture books, the difference between female characters with long and short hair is relatively small. In both groups, tools and toys constituted a larger share of the items carried by male characters than of those carried by female characters, whereas household and decorative items constituted a larger share of the items carried by female characters.

### 3.2. Gender Differences in Character Activities and Social Identity

#### 3.2.1. Character Activities Across Chinese-Authored and Non-Chinese-Authored Picture Books

As shown in Table 5, male characters appear more frequently in outdoor settings across both types of picture books. Chinese-authored picture books feature male characters outdoors more often than non-Chinese-authored picture books. Female characters in Chinese-authored picture books were depicted indoors more frequently than outdoors, whereas the difference between indoor and outdoor representation was smaller for female characters in non-Chinese-authored picture books.

Regarding activity types, both categories of picture books show male characters participating in work, leisure, and learning more frequently than female characters, while female characters engage in housework more often than male characters. Descriptively, this pattern indicates that male characters were represented across a wider range of activities and appeared more frequently in these activities. Within leisure activities, male characters were predominantly engaged in ball games and board games, which are more socially participatory and competitive. Female characters, by contrast, were concentrated in handicrafts, knitting, and artistic pursuits, activities characterized by finer motor skills and artistic expression. Regarding domestic chores, however, male characters were coded less frequently than female characters, reflecting a significant imbalance in household task distribution between genders and perpetuating the traditional gender role perception of women as primary caregivers within the home. Additionally, the gender distribution of social interaction differed somewhat between the two groups. In Chinese-authored picture books, male characters accounted for a larger proportion of social-interaction instances than female characters (19.2% vs. 13.4%). In non-Chinese-authored picture books, the proportions were similar, with female characters accounting for a slightly larger share than male characters (17.0% vs. 16.0%).

#### 3.2.2. Social Identity Across Chinese-Authored and Non-Chinese-Authored Picture Books

Social identity encompassed family and occupational roles. Across both book categories, family identities were coded more often for female characters, whereas occupational identities were coded more often for male characters. Descriptively, among male characters in Chinese-authored picture books, family identities accounted for a larger proportion than occupational identities (65.4% vs. 34.6%), whereas the opposite pattern was observed in non-Chinese-authored picture books (37.5% vs. 62.5%).

Family identity included the roles of “educator”, “housework laborer”, “economic provider”, and “emotional supporter”. As shown in Table 6, family-identity codes were more frequent for female than for male characters. Specifically, in both types of picture books, male characters were coded more often as economic providers than female characters; female characters were coded more often as housework laborers and emotional supporters than male characters. This also corresponds to the coding results for activity types concerning housework. For the educator identity, male characters in Chinese-authored picture books received more codes than female characters. For example, in *Ringing Out the Old Year and Welcoming the New: Spring Festival*, multiple scenes depict male characters (the grandfather) explaining to children the customs of eating dumplings during the Spring Festival and the origins of “observing the New Year’ Eve”. In *Going Back to Grandma’s for the Spring Festival*, activities such as teaching children to write Spring Festival couplets, explaining stories about stamps, and using farm tools are also undertaken by males (grandfather and father). In contrast, female characters were coded more frequently than male characters in this identity category in non-Chinese-authored picture books.

In terms of occupational identity, the total number of occupational-identity codes was higher for male than for female characters. Specifically, male characters had more diverse occupational roles and a broader occupational range, covering fields such as construction, healthcare, agriculture, industry, and business, as well as education. In contrast, female characters’ occupational roles were relatively narrow, often confined to education, services, and the arts. Portrayals of female characters in occupations that challenged traditional gender stereotypes, such as police officers, were relatively rare.

### 3.3. Behavioral Characteristics Across Chinese-Authored and Non-Chinese-Authored Books

A chi-square test of independence was first conducted to examine whether overall character gender representation differed between Chinese-authored and non-Chinese-authored picture books. The association between book type and character gender was significant, χ^2^ (1) = 7.09, *p* = 0.008, Cramer’s V = 0.06. Non-Chinese-authored picture books included a higher proportion of male characters (61.0%) than Chinese-authored picture books (55.4%). Next, chi-square tests of independence were conducted within each book type to examine the association between character gender and behavioral characteristics. The association was significant for Chinese-authored picture books, χ^2^ (3) = 23.46, *p* < 0.001, Cramer’s V = 0.15, and non-Chinese-authored picture books, χ^2^ (3) = 26.60, *p* < 0.001, Cramer’s V = 0.16. A three-way log-linear analysis showed that the interaction among book type, character gender, and behavioral characteristics was not significant, χ^2^ (3) = 3.72, *p* = 0.294, indicating that the gendered distribution of behavioral characteristics did not significantly differ between Chinese-authored and non-Chinese-authored picture books.

Follow-up exact binomial tests were conducted within each behavioral category to determine whether the proportion of male characters differed from female characters, with Holm corrections applied within each book type. In Chinese-authored picture books, male characters were significantly overrepresented in exploratory action, male proportion = 0.65, Cohen’s h = 0.31, *p* < 0.001, control and dominance, male proportion = 0.59, Cohen’s h = 0.18, *p* = 0.022. Gender differences were not significant for emotional interaction, male proportion = 0.47, Cohen’s h = −0.05, *p* = 0.291, or collaborative support, male proportion = 0.58, Cohen’s h = 0.17, *p* = 0.090. In non-Chinese-authored picture books, male characters were significantly overrepresented in exploratory action, male proportion = 0.70, Cohen’s h = 0.42, *p* < 0.001, and collaborative support, male proportion = 0.66, Cohen’s h = 0.32, *p* = 0.001. Gender differences were not significant for emotional interaction, male proportion = 0.54, Cohen’s h = 0.09, *p* = 0.092, or control and dominance, male proportion = 0.52, Cohen’s h = 0.03, *p* = 0.899. No behavioral category showed a statistically significant overrepresentation of female characters.

To complement the inferential analyses, the following results describe the distributional patterns within each coding dimension using frequencies and percentages. As shown in Table 7, in the dimension of emotional interaction, female characters received more emotional-interaction codes than male characters. This pattern may reflect conventional expectations that women should maintain relational bonds through emotional expression, whereas men should remain rational, strong, and emotionally restrained.

In the exploratory action dimension, male characters were more often portrayed as explorers or pioneers, with climbing, running, and jumping serving as typical behavioral markers. However, such actions are rarely seen in females, which obscures the possibility of diverse behaviors such as strength and a desire for exploration in females. Few works in either group attempted to portray female characters as proactive and agentic.

With regard to collaborative and supportive behavior, male characters in both categories of picture books received slightly more codes than their female counterparts. Female characters tended toward emotional support, facilitating task completion or problem-solving through verbal encouragement, guidance, or subtle cues, whereas male characters favored instrumental support, employing tools and concrete actions to advance objectives. This pattern may reflect the long-term sociocultural conditioning of gender roles.

In the dimension of control and dominance, coding results indicate slightly more male-coded instances than female-coded ones in Chinese-authored picture books, while non-Chinese-authored picture books show more female-coded instances than male-coded ones. Specifically, male characters tended toward leadership and command, often initiating events. Female characters, by contrast, showed more directive and controlling behaviors, often by arranging specific tasks.

## 4. Discussion

This study examined gender representation in Chinese-authored and non-Chinese-authored picture books across cover characters, protagonists, physical appearance, character activities, social identity, and behavioral characteristics. Overall, the findings suggest that gender imbalance remains evident in both Chinese-authored and non-Chinese-authored picture books. The following discussion is organized according to the three research questions.

### 4.1. Gender Visibility and Visual Representation in Picture Books

The first research question concerned the overall patterns of gender representation in Chinese-authored and non-Chinese-authored picture books, with particular attention to cover characters, protagonists, and physical appearance. The results show that male characters remained more visible than female characters in both categories of books. Viewed through sociocultural theory and gender-schema theory, this imbalance concerns not only visual visibility but also the repeated association of masculinity with narrative centrality.

This finding is consistent with previous studies showing that male characters appear more frequently than female characters in book titles, illustrations, and central narrative roles (Casey et al., 2021; Hamilton et al., 2006; Huang et al., 2025; Lindsay, 2024; Tepper & Cassidy, 1999; Turner-Bowker, 1996; M. Wang & Lee, 2024). At the narrative level, these patterns suggest the continued prominence of male characters. Male characters are more often positioned as the agents who drive the storyline, whereas female characters are more likely to appear in peripheral or secondary roles. The present study did not directly examine children’s interpretations or responses; however, previous research suggests that repeated exposure to such patterns may contribute to children’s perceptions of who is represented as central, active, and narratively important (Ismatullina et al., 2022; Saba et al., 2023; Song et al., 2024).

In terms of physical appearance, the descriptive analysis indicated some differences between Chinese-authored and non-Chinese-authored picture books. Chinese-authored picture books tended to rely more heavily on conventional gender markers. Long hair and dresses were frequently used as visual signs of femininity, reinforcing recognizable distinctions between male and female character appearances. This pattern is consistent with previous studies showing persistent gender stereotyping in children’s picture books, particularly in relation to hairstyles, clothing, accessories, and gendered visual symbols (Cai, 2013; Chu, 2020; Ji & Zhang, 2021; Tang, 2020). Consistent with gender-schema theory, these recurring visual cues make binary gender categories readily recognizable while restricting the visible range of gender expression.

By contrast, non-Chinese-authored picture books appeared to present somewhat greater variation in female characters’ hairstyles and clothing within the sampled corpus. This difference may be related to broader cultural and historical contexts. From a sociocultural perspective, this pattern may reflect differences in the historical and institutional contexts of book production, including the influence of feminist movements and sustained critiques of gender stereotypes in some source countries. Previous studies have shown that gender representation in children’s books changes alongside broader social movements and scholarly attention to gender equality (Clark et al., 2003; Gooden & Gooden, 2001; McCabe et al., 2011). However, this does not mean that non-Chinese-authored picture books are free from stereotypes. Because the physical-appearance analysis was descriptive and the non-Chinese-authored books represented heterogeneous cultural origins, these patterns should not be interpreted as evidence of a general cultural effect.

### 4.2. Gendered Activities, Spatial Positioning, and Social Identity

The second research question asked how activities and social identities were distributed across male and female characters in Chinese-authored and non-Chinese-authored picture books. The findings suggest that gender stereotypes were present in both activity spaces and social roles, although the observed patterns differed to some extent between the two categories of books.

In Chinese-authored picture books, male characters appeared more frequently in outdoor and public spaces, whereas female characters were more often associated with indoor and family-related contexts. This finding is consistent with previous studies showing that male characters in children’s books are more likely to be represented in outdoor settings (Chu, 2020; Hamilton et al., 2006; Qin et al., 2018). From sociocultural and gender-schema perspectives, this distribution reproduces a public–private distinction in which male characters are associated with exploration and social participation and female characters with care and domesticity. Using Schwartz’s framework as an auxiliary interpretive lens, the association of male characters with exploration, initiative, and participation in public life may correspond to values of autonomy and mastery, whereas the association of female characters with family and relational roles may correspond to embeddedness. This interpretation is conceptual rather than empirical, because the present study did not directly measure cultural values. By contrast, non-Chinese-authored picture books showed relatively smaller descriptive gender differences in activity spaces within the sampled corpus.

A similar pattern appeared in the representation of social identity. Male characters were associated with a wider range of occupations, whereas female characters were often limited to a narrower set of traditionally feminine occupations. This finding is consistent with previous studies showing that occupational stereotypes in picture books have remained persistent over time (Hale, 2024; Hamilton et al., 2006; M. Wang & Lee, 2024). From a gender-schema perspective, recurring occupational distributions may reinforce associations between gender and socially appropriate work. Through Schwartz’s hierarchy–egalitarianism dimension, they may also be interpreted as unequal symbolic access to socially valued roles, although cultural values were not measured directly. Previous research suggests that repeated exposure to gender-stereotyped occupations may contribute to children’s assumptions about gender-appropriate careers (Peterson & Lach, 1990). Likewise, portraying female characters primarily in supportive or caring occupations could narrow the range of professional identities made visible to young readers (Jalalkamali & Doratli, 2022; Song et al., 2024). These patterns may not fully reflect the increasing diversity of women’s employment in contemporary society, although this comparison is contextual rather than directly equivalent.

Many studies have redefined fatherhood by emphasizing fathers’ caregiving responsibilities and diverse parenting capacities (Adams et al., 2011; Pleck, 1981; Silverstein, 1996), and have documented increased paternal participation in family caregiving under shifting work–family conditions (Augustine & Prickett, 2022; Couch et al., 2022; Cutler & Lewis, 2024). The present coding did not distinguish fathers from other male family members and therefore does not permit fatherhood to be assessed separately. However, the present study’s coding findings showed that male characters’ presence in housework labor and emotional support was lower than that of female characters. Only a small number of picture books portray male characters as prioritizing family companionship and emotional communication, such as *Xiao Di and the Tearful Orange*, *Shopping at the Bazaar with Dad, Mitchell’s License,* and *Something from Nothing*. From sociocultural and gender-schema perspectives, this uneven distribution indicates that family responsibilities remain organized through conventional gender categories in the sampled books. Recognizing this gap between social reality and literary representation is important. Previous research suggests that, during socialization, children form expectations about parental roles, while adults also construct their own parenting identities through available cultural models (Anderson & Hamilton, 2005).

### 4.3. Behavioral Characteristics and Gendered Agency

The third research question focused on the behavioral characteristics associated with male and female characters. The findings suggest that gender stereotypes are not only reflected in visual symbols and social roles, but also in the representation of agency and behavioral tendencies.

The analyses showed that character gender distributions differed between the two book categories, while gendered patterns in behavioral characteristics remained evident within both. Follow-up binomial tests further showed that male characters were significantly overrepresented in exploratory action in both book categories. Across the two categories, male characters were more often associated with adventure, initiative, exploration, and problem solving, whereas female characters were more frequently linked with care, emotion, support, and relational responsibility. These findings are consistent with previous studies showing that behavioral representations in picture books remain closely connected to traditional gender norms (Chu, 2020; Hamilton et al., 2006; Ji & Zhang, 2021; Tang, 2020). From a gender-schema perspective, these recurring associations may reinforce schemas linking masculinity with agency and femininity with caregiving. Sociocultural theory situates these patterns within culturally available models of gendered behavior. As an auxiliary interpretive lens, Schwartz’s framework suggests that male portrayals may symbolically align with autonomy and mastery, whereas female portrayals may align more closely with embeddedness and relational responsibility; however, these patterns should not be interpreted as direct measures of cultural-value orientations.

In Chinese-authored picture books, male characters were also significantly overrepresented in control and dominance, a pattern consistent with conventional associations between masculinity, authority, and leadership. In non-Chinese-authored picture books, male characters were significantly overrepresented in collaborative support, indicating that supportive behavior was not represented as exclusively feminine. No behavioral category in either book type showed a significant overrepresentation of female characters. These results suggest that gendered agency was expressed differently across the two categories but remained oriented primarily toward male overrepresentation. From a sociocultural perspective, these differences may reflect distinct cultural repertoires of acceptable male behavior; however, from a gender-schema perspective, both patterns continue to position male characters as prominent social agents. Schwartz’s framework further highlights a symbolic contrast between mastery and hierarchy on the one hand and collaborative or egalitarian orientations on the other.

However, both Chinese-authored and non-Chinese-authored picture books included works that attempted to challenge traditional gender frameworks. The following cases illustrate possible forms of female agency within the corpus. In *The Secret in the Book*, the girl enters an imaginative world, but her actions remain partly shaped by external guidance. In *Journey*, the girl initiates the narrative by creating a passage into a fantasy world and acting independently within it. These individual works were selected as illustrative cases after the corpus-level patterns had been identified and should not be regarded as representative of either book category. Viewed through Schwartz’s autonomy and mastery orientations, these cases illustrate different forms of self-directed agency rather than cultural-value scores. This comparison suggests that gender representation should not be evaluated solely through the number of female protagonists; the degree of autonomy, decision-making power, and narrative agency granted to female characters is also relevant.

## 5. Implications

The findings of this study yield several practical implications. First, because female characters were less visible in central roles and were represented within a narrower range of occupational and family identities, picture-book authors may benefit from reflecting more critically on gender equality and developing multidimensional characters whose traits and roles are not constrained by sex. Given that previous research has likewise documented the underrepresentation of female characters in central roles (Hamilton et al., 2006; McCabe et al., 2011), authors could consider more balanced gender representation. Award bodies may also consider incorporating gender representation into existing literary and artistic evaluation criteria, supported by diverse judging panels and feedback on recurring stereotyped patterns. In light of the patterns identified in the corpus and prior literature, publishers, as final gatekeepers, could give greater attention to gender-equitable content when selecting and promoting books. Because authors write from culturally situated positions, Chinese-authored picture books may present social settings and role expectations that are more immediately familiar to Chinese children, making them useful starting points for classroom teaching and parent–child discussion. Non-Chinese-authored picture books may offer different cultural perspectives and alternative representations of gender roles. Thus, the two groups provide complementary rather than hierarchically different educational resources.

Second, because gender differences were observed in appearance, activities, occupational roles, caregiving, and behavioral characteristics, adult mediation may be valuable during reading. Parents and educators, as primary selectors of children’s reading materials, could prioritize books featuring varied roles and discuss stereotyped representations when they arise, as access to gender-equitable books has been associated with more flexible gender attitudes in young children (Diekman & Murnen, 2004). Consistent with a sociocultural perspective, adults can help children interpret how particular representations are embedded in specific cultural contexts. When reading non-Chinese-authored picture books, additional contextual guidance may be especially useful in helping children distinguish culturally specific role expectations from assumptions about gender that are treated as universal. During shared reading, adults may use the interplay of text and images to discuss how gender is associated with particular occupations and behaviors and connect these representations with children’s everyday observations. Such discussions may help children consider that abilities and social roles are not inherently determined by gender (Kollmayer et al., 2018). Inviting children to rewrite plots or redesign characters may also provide opportunities for critical reflection (Martin & Ruble, 2004). The practical value of these approaches should be further examined through intervention-based research.

## 6. Limitations

First, this study employed a descriptive content-analysis design. Coding instances were clustered within characters and books, and some tables used different analytical denominators. Inferential analyses were therefore limited to selected dimensions for which book-level comparisons were appropriate, whereas the remaining dimensions were analyzed descriptively. No formal diversity index was calculated. The observed differences characterize the sampled corpora and should not be generalized as population- or culture-level effects.

Second, the corpus of non-Chinese-authored picture books included books from multiple countries, languages, publication periods, and cultural traditions. Because source groups were uneven and some subgroup cells were small, cultural origin could not be systematically disaggregated across all dimensions. The distribution of Chinese-authored and non-Chinese-authored books across the three sampling sources may also have influenced the findings. Excluding animal protagonists and books without identifiable binary gender cues may have reduced the representation of less explicitly gender-marked stories.

Third, the coding scheme classified characters only as male or female and therefore could not represent nonbinary, gender-fluid, or deliberately ambiguous identities. The study examined books rather than children, caregivers, classrooms, or reading interactions. Cultural values and social axioms were used as interpretive frameworks rather than directly measured variables. Accordingly, conclusions concerning readers’ beliefs, development, adult mediation, or psychological effects require separate child-level, audience-based, and cross-cultural research.

## 7. Conclusions

This study examined gender representations in Chinese-authored and non-Chinese-authored picture books for young children through a content analysis of 148 selected works. The findings suggest that gender stereotypes remain present in both types of picture books, which continue to reflect traditional gender expectations and binary gender frameworks. These results indicate that picture books are not only literary and educational materials but also important cultural resources through which children may encounter social meanings of gender. Therefore, authors, publishers, parents, and educators should pay greater attention to the gender messages embedded in picture books and provide children with more diverse and equitable representations of gender. Future research may further explore how children interpret these gender representations during actual reading practices and how adult–child interactions shape children’s gender-role understanding.

## Figures and Tables

**Table 1:** Coding Scheme.

Primary Coding	Secondary Coding
**Patterns of gender representation**	
Cover Character	Male Character, Female Character, Both Male and Female Present, No Gender Feature
Protagonist	Male protagonist, Female protagonist, Both Male and Female protagonist, No Gender Feature
Physical Appearance	Clothing, Hairstyle, Carried Items
**Activities and Social Identity**	
Activities	Settings, Types
Social Identity	Family Identity, Occupational Identity
**Behavioral Characteristics**	Emotional Interaction, Exploratory Action, Collaborative Support, Control and dominance

**Table 2:** Percentage of Gender Role Representations on the Covers of Chinese-authored (*n* = 79) and Non-Chinese-authored Chinese Picture Books (*n* = 69).

	Originality	Chinese-Authored Picture Books	Non-Chinese-Authored Picture Books
Categories			
Male		32.9% (26)	43.5% (30)
Female		27.8% (22)	27.5% (19)
Both male and female		26.6% (21)	21.7% (15)
No gender feature		12.7% (10)	7.2% (5)
Total		100% (79)	100% (69)

Note: Values are presented as percentages, with frequencies in parentheses. Percentages may not sum to 100% because of rounding; the same applies to the tables below.

**Table 3:** Gender Distribution of Protagonists in Chinese-authored (*n* = 79) and Non-Chinese-authored Picture Books (*n* = 69).

	Originality	Chinese-Authored Picture Books	Non-Chinese-Authored Picture Books
Category			
Male		50.6% (40)	58.0% (40)
Female		32.9% (26)	27.5% (19)
Both male and female		12.7% (10)	4.3% (3)
No gender feature		3.8% (3)	10.1% (7)
Total		100% (79)	100% (69)

**Table 4:** Nodes Related to Physical Appearance in Chinese-authored (*n* = 79) and Non-Chinese-authored Picture Books (*n* = 69).

Categories		Chinese-Authored Picture Books		Non-Chinese-Authored Picture Books	
		Male	Female	Male	Female
Clothing	Trousers	100% (125)	39.8% (45)	100% (162)	34.8% (49)
	Skirts	0% (0)	60.2% (68)	0% (0)	65.2% (92)
	Total	100% (125)	100% (113)	100% (162)	100% (141)
	Low lightness	51.5% (87)	36.1% (60)	55.6% (95)	35.0% (50)
	High lightness	48.5% (82)	63.9% (106)	44.4% (76)	65.0% (93)
	Total	100% (169)	100% (166)	100% (171)	100% (143)
Hairstyle	Long Hair	0% (0)	92.9% (105)	1.9% (3)	53.7% (72)
	Short Hair	100% (103)	7.1% (8)	98.1% (154)	46.3% (62)
	Total	100% (103)	100% (113)	100% (157)	100% (134)
Carried Items	Tools	46.2% (49)	9.6% (9)	50.0% (81)	30.5% (39)
	Household items	1.9% (2)	27.7% (26)	2.5% (4)	20.3% (26)
	Decorations	31.1% (33)	43.6% (41)	37.7% (61)	42.2% (54)
	Toys	20.8% (22)	19.1% (18)	9.9% (16)	7.0% (9)
	Total	100% (106)	100% (94)	100% (162)	100% (128)

Note: Percentages were calculated within each coding dimension using the total number of coded instances as the denominator. For clothing, style (trousers and skirts) and color (low- and high-lightness) were calculated separately. The same calculation applies to the subsequent tables.

**Table 5:** Nodes Related to Activity Setting and Activity Types in Chinese-authored (*n* = 79) and Non-Chinese-authored Picture Books (*n* = 69).

Categories		Chinese-Authored Picture Books		Non-Chinese-Authored Picture Books	
		Male	Female	Male	Female
Setting	Indoor	21.2% (14)	57.1% (36)	38.9% (42)	47.7% (41)
	Outdoor	78.8% (52)	42.9% (27)	61.1% (66)	52.3% (45)
	Total	100% (66)	100% (63)	100% (108)	100% (86)
Types	Work	20.8% (26)	14.8% (21)	24.0% (24)	14.9% (14)
	Housework	12.0% (15)	47.2% (67)	8.0% (8)	22.3% (21)
	Social interaction	19.2% (24)	13.4% (19)	16.0% (16)	17.0% (16)
	Leisure	41.6% (52)	21.1% (30)	45.0% (45)	40.4% (38)
	Learning	6.4% (8)	3.5% (5)	7.0% (7)	5.3% (5)
	Total	100% (125)	100% (142)	100% (100)	100% (94)

**Table 6:** Nodes Related to Social Identities in Chinese-authored (*n* = 79) and Non-Chinese-authored Picture Books (*n* = 69).

Categories		Chinese-Authored Picture Books		Non-Chinese-Authored Picture Books	
		Male	Female	Male	Female
**Family Identity**	Educator	13.1% (14)	6.1% (9)	6.9% (5)	20.0% (14)
	Housework laborer	12.1% (13)	45.3% (67)	9.7% (7)	31.4% (22)
	Economic provider	12.1% (13)	5.4% (8)	2.8% (2)	0% (0)
	Emotional supporter	28.0% (30)	31.1% (46)	18.1% (13)	25.7% (18)
	Total	65.4% (70)	87.8% (130)	37.5% (27)	77.1% (54)
**Occupation Identity**		34.6% (37)	12.2% (18)	62.5% (45)	22.9% (16)
Total		100% (107)	100% (148)	100% (72)	100% (70)

**Table 7:** Nodes Related to Behavioral Characteristics in Chinese-authored (*n* = 79) and Non-Chinese-authored Picture Books (*n* = 69).

Behavioral Characteristics	Chinese-Authored Picture Books	Chinese-Authored Picture Books	Non-Chinese-Authored Picture Books	Non-Chinese-Authored Picture Books
	Male	Female	Male	Female
Emotional Interaction	36.8% (225)	50.5% (249)	43.2% (289)	56.5% (242)
Exploratory Action	26.8% (164)	17.8% (88)	37.7% (252)	24.8% (106)
Collaborative support	14.9% (91)	13.2% (65)	14.3% (96)	11.7% (50)
Control and Dominance	21.6% (132)	18.5% (91)	4.8% (32)	7.0% (30)
Total	100.0% (612)	100.0% (493)	100.0% (669)	100% (428)

## Data Availability

The picture books analyzed in this study were primarily obtained through physical borrowing and digital resource databases. All picture books were independently verified by two researchers to confirm the accuracy of edition information and content. The coding data and related analytical materials generated using NVivo 15.0 for the 148 picture books in this study are available from the corresponding author upon request.

## Appendix A

**Table A1:** Analysis Checklist of Chinese-authored Picture Books.

No.	Book Title	No.	Book Title
1	Little Li Fu the Crybaby (爱哭的小立甫)	41	Grandma, I Love You (外婆，我爱你)
2	“Baby, I Understand You” Picture Book Series: The Amazing Luo En (“宝贝，我懂你”系列图画书 了不起的罗恩)	42	My Dad Is a Soldier (我爸爸是军人)
3	Dad’s Train (爸爸的火车)	43	I Can Protect My Eyes (我会保护眼睛)
4	The Secrets of Terra-Cotta Warriors (兵马俑的秘密) *	44	I Might Become a Painter (我可能会成为一个画家)
5	Don’t Skip Rope with Frogs (不要和青蛙跳绳)	45	Who Am I? (我是谁)
6	Ringing Out the Old Year and Welcoming the New: Spring Festival (辞旧迎新过大年——春节)	46	I Want a Little Brother (我想有个弟弟)
7	When Fingers Dance (当手指跳舞时)	47	I Still Love You (我依然爱你)
8	Play with Me (和我玩吧)	48	I Have a Dream (我有一个梦)
9	The “Odd” Boy and His Wordless Picture Book (“怪”男孩和他的无字书)	49	Wash My Hands Clean (洗洗小手好干净)
10	Homecoming (回家)	50	Xiao Di and the Tearful Orange (小笛和流泪的橘子)
11	Going Back to Grandma’s for Spring Festival (回老家过年)	51	An Zai Will Get Better (安仔一定会变好)
12	Back to the Countryside (回乡下)	52	A New Year’s Reunion (团圆) *
13	The Gold-Medal Postman (金牌邮递员)	53	Grandpa’s 14 Games (爷爷的14个游戏) *
14	A Journey of 9000 Millimeters (九千毫米的旅行) *	54	Grandpa’s Teeth, My Teeth (爷爷的牙，我的牙)
15	There’s Something I Can’t Buy (有什么东西我买不来)	55	A Small One, A Big One (一条小小的，一块大大的)
16	Laba Porridge (腊八粥)	56	Yi Wazi (翼娃子)
17	Grandma’s Braised Pork (姥姥的红烧肉)	57	What Will I do When I Grow Up? (长大以后干什么？)
18	Amazing Vegetables (了不起的蔬菜)	58	The Mid-Autumn Festival (中秋节)
19	Come On, Mom! (妈妈，加油！)	59	The Garden in the Bamboo Basket (竹篮里的花园)
20	Mom’s Heart, Mom’s Tree (妈妈心 妈妈树)	60	My Favorite Day (最喜欢的一天)
21	Slow Yi Di (慢吞吞的易迪)	61	Xixi (西西)
22	Those Years, That City (那些年 那座城)	62	The King of Hide and Seek (躲猫猫大王) *
23	Nana Cleans the Room (娜娜打扫房间)	63	The Morning Market at Lotus Town (荷花镇的早市) *
24	Grandma’s Qipao (奶奶的旗袍)	64	Me and My Bike (我和我的脚踏车) *
25	Your Hand, My Hand, His Hand (你的手我的手他的手)	65	Enchanted by Opera (迷戏)
26	Niuniu’s Happy Day (妞妞的幸福一天)	66	The Scariest Day (最可怕的一天)
27	Going on an Adventure (去冒险)	67	Teeth, Teeth, Throw Them on the Roof (牙齿，牙齿，扔屋顶)
28	The Secret in the Book (书里的秘密)	68	Where Does Rice Come From? (盘中餐) *
29	Water! (水哎)	69	Where Did the Ticket Go? (车票去哪里了)
30	Dark clouds, Coming Rain (天黑黑要落雨)	70	Going to the Zoo Together (一起去动物园)
31	Little Bear, Run Fast! (小熊，快跑)	71	Happy Birthday (生日快乐)
32	I Know You’re All Still Awake (我知道你们都没睡觉)	72	Making trouble for Mom (给妈妈捣乱)
33	Dad Goes to Work (爸爸去上班)	73	A Bicycle (一辆自行车)
34	That Deep Blue Bird Is My Daddy (那只深蓝色的鸟是我爸爸) *	74	Riddles (谜语)
35	Granny Mian Can’t Sleep (棉婆婆睡不着)	75	There’s Always a Reason to Eat Baozi (总有一个吃包子的理由)
36	Diandian’s Summer (点点的夏天)	76	Playing with Lanterns (打灯笼) *
37	What’s in Grandpa’s Bag? (外公的包里有什么？)	77	Can I Skip School Today? (今天，我可以不上学吗？)
38	The Talking Hand (会说话的手)	78	The Big-Footed Girl (大脚姑娘)
39	Square-Faced Grandpa and Round-Faced Grandma (方脸公公和圆脸婆婆)	79	Shopping at the Bazaar with Dad (我和爸爸逛巴扎)
40	Summer Night Concert (夏日音乐会) *		

Note: Picture books marked with an asterisk have corresponding officially published English versions; picture books without an asterisk do not have corresponding officially published English versions, and their English titles were translated by the author of this article.

**Table A2:** Analysis Checklist of Non-Chinese-authored Picture Books.

No.	Book Title	No.	Book Title
1	Grandpa Ai and the bear on the roof (艾爷爷和屋顶上的熊) *	36	Daniel Finds a Poem (丹尼尔找到一首诗) *
2	No, David! (大卫，不可以！) *	37	When Sophie Gets Angry—Really, Really Angry… (菲菲生气了) *
3	Pedro’s Whale (多多的鲸鱼) *	38	Mr. Gumpy’s Outing (和甘伯伯去游河) *
4	Sticky Superman (黏黏超人) *	39	The Whale (鲸鱼)
5	What Happens at Night (谁知道夜里会发生什么) *	40	The Tiger Who Came to Tea (老虎来喝下午茶) *
6	My Dad (我爸爸) *	41	The Growing Story (妈妈，我什么时候长大)
7	My Friend with Autism (我的孤独症朋友) *	42	Library Lion (图书馆狮子) *
8	I Just Want to Say Good Night(正要说晚安) *	43	Where’s Our Mama? (我们的妈妈在哪里) *
9	Red Red Red (啊——我生气了) *	44	We ‘re Going on a Bear Hunt (我们要去捉狗熊) *
10	Barbapapa (巴巴爸爸经典故事系列：巴巴爸爸的诞生) *	45	When My Hair Grows Long… (小真的长头发) *
11	The Baby Tree (宝宝树) *	46	If You Give a Mouse a Cookie (要是你给老鼠吃饼干) *
12	The First Time I Slept by Myself (第一次自己睡觉)	47	Joseph Had a Little Overcoat (约瑟夫有件旧外套) *
13	The Hole (洞)	48	Boy in the Moon (月光男孩) *
14	Sid the Signal (红绿灯眨眼睛) *	49	Emma (艾玛画画) *
15	The Carrot Seed (胡萝卜的种子) *	50	Journey (不可思议的旅程) *
16	It’s My Birthday (今天是我的生日) *	51	John Patrick Norman McHennessy: The boy who was always late (迟到大王) *
17	The Blue Balloon (蓝气球) *	52	Town is by the tea (等爸爸回家) *
18	Mitchell’s License (米琪的驾照) *	53	My First Errand (第一次上街买东西)
19	Do You Love…? (你喜欢…)	54	La grande question (大问题) *
20	Are You Awake? (你醒了吗？) *	55	Miss Rumphius (花婆婆) *
21	Mottainai Grandma (怕浪费婆婆)	56	Clocks and More Clocks (金老爷买钟) *
22	Pengpeng Loves Stamping, Bang! (朋朋爱盖章，砰！)	57	Stone Soup (石头汤) *
23	Ella Sarah Gets Dressed (萨拉就要这样穿) *	58	A Tree Is Nice (树真好) *
24	What’s That Stinky Smell? (什么这么臭)	59	Sugar Ball (糖球)
25	Blackout (停电以后) *	60	Aldo (我的秘密朋友叫阿德)
26	Still Stuck! (脱不下来啦) *	61	My Mom (我妈妈) *
27	I’m Not Afraid of Shots! (我不怕打针)	62	Nothing (我要买“什么都没有”) *
28	I Won’t Give Up My Rubber Band (我的橡皮筋，不给你) *	63	I Can Explain (我真的有理由) *
29	My Mouth Is a Harmonica (我的嘴巴是一只口琴) *	64	The Snowman (雪人) *
30	I Walked Straight and Straight (我一直一直朝前走)	65	Something from Nothing (爷爷一定有办法) *
31	One Summer’s Day (夏日的一天)	66	Cloudy With a Chance of Meatballs (阴天有时下肉丸) *
32	The Ever-Growing Leg (长个不停的腿) *	67	Courage (勇气) *
33	10 Minutes till Bedtime (再过10分钟就睡觉) *	68	Moon Man (月亮先生) *
34	The Round Wheel (圆圆的轮子)	69	The Night Walk (走在星空下) *
35	A Magic Line (一条神奇的线)		

Note: Picture books marked with an asterisk have corresponding officially published English versions; picture books without an asterisk do not have corresponding officially published English versions, and their English titles were translated by the author of this article.
